# mHealth text and voice communication for monitoring people with chronic diseases in low-resource settings: a realist review

**DOI:** 10.1136/bmjgh-2017-000543

**Published:** 2018-03-06

**Authors:** Jocelyn Anstey Watkins, Jane Goudge, Francesc Xavier Gómez-Olivé, Caroline Huxley, Katherine Dodd, Frances Griffiths

**Affiliations:** 1 Division of Health Sciences, Warwick Medical School, University of Warwick, Coventry, UK; 2 Centre for Health Policy, Faculty of Health Sciences, School of Public Health, University of the Witwatersrand, Johannesburg, South Africa; 3 MRC/Wits Rural Public Health and Health Transitions Research Unit (Agincourt), School of Public Health, Faculty of Health Sciences, University of the Witwatersrand, Johannesburg, South Africa

**Keywords:** systematic review, health systems

## Abstract

**Background:**

Routine monitoring by patients and healthcare providers to manage chronic disease is vital, though this can be challenging in low-resourced health systems. Mobile health (mHealth) has been proposed as one way to improve management of chronic diseases. Past mHealth reviews have proposed the need for a greater understanding around how the theoretical constructs in mHealth interventions actually work. In response, we synthesised evidence from primary studies on monitoring of chronic diseases using two-way digital text or voice communication between a patient and health worker. We did this in order to understand the important considerations for the design of mHealth interventions.

**Method:**

Articles retrieved were systematically screened and analysed to elicit explanations of mHealth monitoring interventions. These explanations were consolidated into programme theory and compared with existing theory and frameworks. We identified variation in outcomes to understand how context moderates the outcome.

**Results:**

Four articles were identified—monitoring of hypertension and HIV/AIDS from: Kenya, Pakistan, Honduras and Mexico and South Africa. Six components were found in all four interventions: reminders, patient observation of health state, motivational education/advice, provision of support communication, targeted actions and praise and encouragement. Intervention components were mapped to existing frameworks and theory. Variation in outcome identified in subgroup analysis suggests greater impact is achieved with certain patient groups, such as those with low literacy, those with stressful life events or those early in the disease trajectory. There was no other evidence in the included studies of the effect of context on the intervention and outcome.

**Conclusion:**

mHealth interventions for monitoring chronic disease in low-resource settings, based on existing frameworks and theory, can be effective. A match between what the intervention provides and the needs or social factors relevant to specific patient group increases the effect. It was not possible to understand the impact of context on intervention and outcome beyond these patient-level measures as no evidence was provided in the study reports.

**Key questions**

**What is already known about this topic?**

- The burden of chronic diseases is an escalating problem globally. Evidence reviews suggest mHealth interventions delivered in low-income and middle-income countries can be effective in improving health outcome for people living with chronic disease.

**What are the new findings?**

- Effective interventions using two-way digital communication between healthcare providers and patients living with long-term conditions are based on established frameworks and behaviour change theory.
- Outcome is improved for patients with low literacy, stress-inducing life events and being recently diagnosed.
- Contextual factors beyond those measured at patient level were not reported in the studies.

**Recommendations for policy**

- mHealth interventions for improving the monitoring of chronic disease, when based on existing frameworks and theory, have potential for improving patient care and health outcome, particularly when tailored to the needs of specific patient groups.

## Introduction

The burden of chronic diseases is an escalating problem in low-income and middle-income countries (LMICs).^1^ The Sustainable Development Goals state that by 2030, improving the prevention and management of chronic communicable and non-communicable diseases is a priority for primary care in public health systems.^2 3^ Chronic diseases are long-term, disruptive and often intrusive to individuals’ everyday lives.^4^ The management of chronic diseases is a dynamic process that varies over time, depending on the disease aetiology and physiology.^5^ Chronic disease involves regular self-care^6 7^ and routine monitoring by patients and health workers to check disease progress or regress.^8^ Long-term monitoring encompasses adhering to treatment and capturing vital signs or clinical indicators. The purpose is to improve outcomes and quality of life^9^ by reducing acute exacerbations and premature death^10^ and to maximise health.

The monitoring of chronic diseases can be challenging, particularly in low-resourced health systems limited by long distances to health facilities and low staff capacity.^11^ In this environment, mobile health (mHealth) has been proposed as an approach to improve management of chronic diseases 12-14 including assistance with monitoring. mHealth technologies used in LMIC include portable wireless devices, including mobile phones and tablets.^15^ mHealth can involve one-way or two-way communication between the health worker and patient, using any digital channel that allows the users to be mobile. Vasudevan *et al*^16^ suggest that strides are being made in strengthening the global mHealth evidence base along with the key ‘best practices’ in scaling mHealth for achieving universal health coverage.

A systematic search for mHealth reviews in seven databases found several mHealth reviews that included aspects of monitoring such as adherence studies,^17^ behaviour change^18^ and attendance reminders.^19 20^ Review authors described how the effectiveness of mHealth evidence is mixed. They suggest there is a lack of understanding of why mHealth interventions for chronic disease management should work (or not) in LMICs.^17 18 20–27^ One of these reviews proposed that future studies should explicitly describe the theoretical constructs that mHealth interventions are targeting.^28^ They argue this will make it clearer how and why the intervention is intended to work and in what circumstances.^28^ A stronger theoretical understanding is likely to strengthen the mHealth evidence base.^27^ However, this search revealed that there is no systematic review set in LMICs specifically focusing on one-way and two-way mHealth communication for monitoring of chronic disease.

This paper responds to the challenge identified in the systematic reviews, by examining the theoretical foundations and mechanisms by which mHealth interventions work (or not), to support chronic disease monitoring in LMICs.

We focus on understanding how monitoring of chronic diseases may be improved using two-way digital communication between a patient and health worker (community health worker/nurse/doctor) in a low-resourced setting to potentially guide future intervention design.

## Method

### The realist method and initial search

A ‘realist review’, also known as a ‘realist synthesis’, makes sense of heterogeneous evidence about complex interventions applied in diverse contexts.^29 30^ It draws on substantive theory and empirical research from across multiple disciplines^31^ to understand how interventions work and for whom. By using the methods of a realist review,^32–34^ we questioned what works, compared with what, how well, with what exposure, with what behaviours, for how long, for whom, in what settings and why^35^? We went through the following realist stages: (1) identifying programme theory, how the study authors intended their interventions to work; (2) testing this programme theory against empirical evidence and established high-level theory and (3) providing guidance for future intervention development.

**Figure 1:**
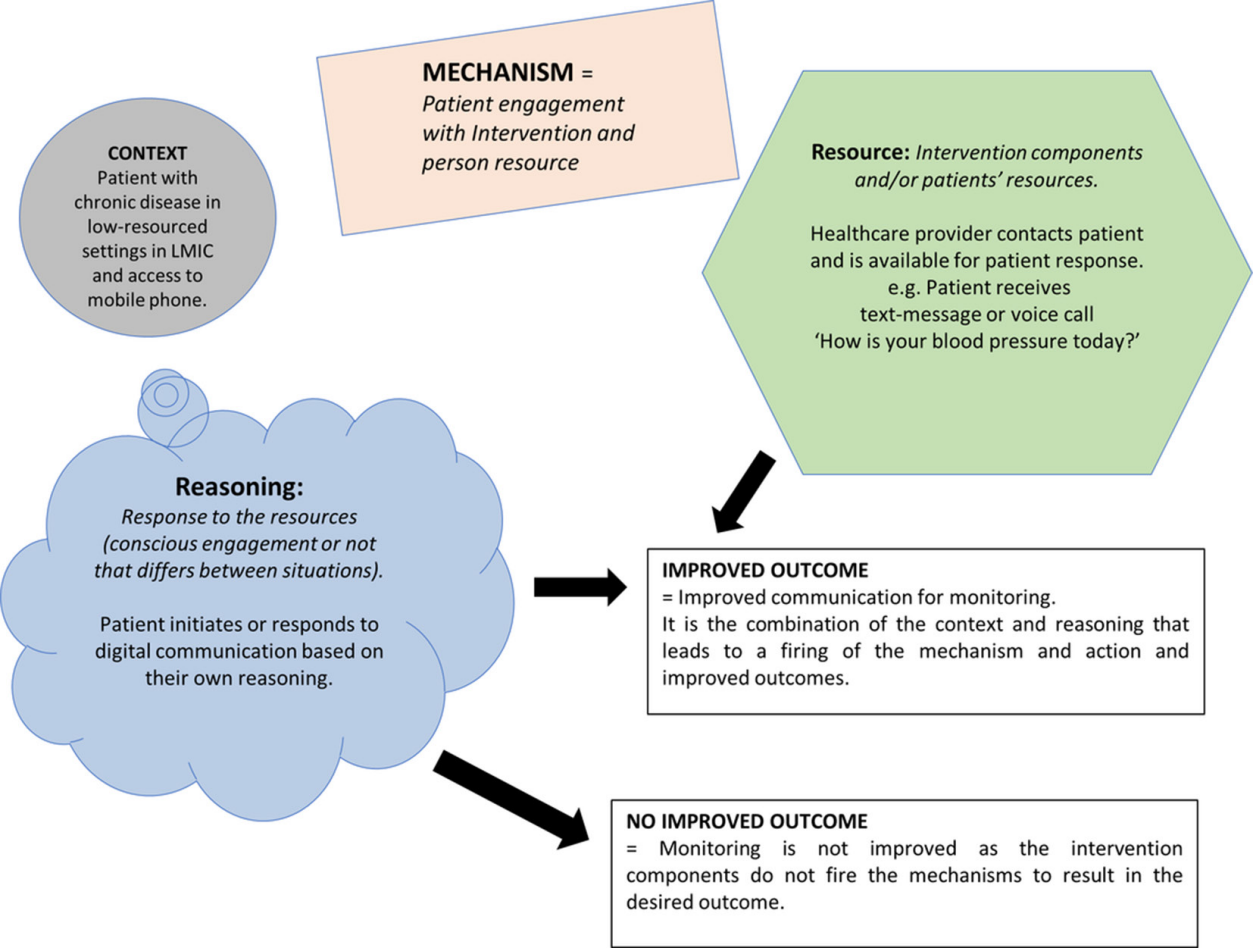
mHealth example of a CMOc summarised from the reviews. CMOc, Context-Mechanism-Outcome configuration; LMIC, low-income and middle-income countries.

We did a preliminary systematic search for systematic reviews of mHealth. From the 10 relevant reviews identified,^17 18 20–27^ we extracted examples of how the review authors thought the interventions worked from their included papers. We mapped these on to the layout of the realist Context-Mechanism-Outcome configuration (CMOc) and adapted the diagram designed by Dalkin^36^ (figure 1) using the example of mHealth monitoring. Figure 1 illustrates how mechanisms are defined as the reactions or responses to the resources available within an interaction process that leads to outcomes.^37^ Mechanisms are the responses to the intervention or patients’ resources and are contingent and conditional, only firing in particular contexts.^38^ The outcomes are the desired response to the resources resulting from the participants’ reasoning^39^ within a particular context.

### Main search to identify empirical papers

To identify relevant empirical studies, we conducted the main search to systematically find papers on the monitoring of chronic disease using two-way text message or voice call interventions in LMICs, written in English, published from January 2000 to March 2017. We limited the search to the year 2000 or later as mobile phones were not widely available before then.^28^ Seven appropriate databases and 12 search terms were used. Titles and abstracts were screened against the inclusion and exclusion criteria, as detailed in table 1. Each reference list of the final included papers in this review was checked to identify further relevant primary research studies.

**Table 1:** Databases, search terms and inclusion and exclusion criteria for identifying empirical studies

Databases searched	Cochrane Register of Controlled Trials, Medline (Ovid), PubMed, Web of Science, Psych Info, Google Scholar, Knowledge for Health
Search terms	Patient* AND low- and middle- income countr* OR LMIC OR developing* AND chronic disease* AND mobile phone* OR text messag* OR SMS OR voice AND monitor* OR self-monitor* OR self-manage*
Inclusion criteria	*Population:* Patients with any chronic disease and all cadres of health workers in a public health system. *Context:* Low-resourced settings of any LMICs. *Intervention design:* The study interventions include two-way exchange of digital information necessary for monitoring initiated by the patient or health worker. The mHealth resource is either voice call or text message based mobile phone communication to improve monitoring of chronic disease. *Monitoring*: monitoring of side effects of medication, monitoring of physiological measures such as blood pressure or monitoring of how well the patient feels and offering relevant information and support and reminding them about future appointments or medication compliance. *Study design:* Empirical research evaluating the effectiveness of the intervention (any design). The study includes a description of the intervention. *Outcomes:* Impact/health and process outcomes (primary and secondary).
Exclusion criteria	Studies set in high-income countries. Studies using only landline telephones or computer-based communication. Protocols or reports of intervention development with no published evidence outcome. Studies not published in English. Feasibility and pilot studies with no outcome for the intervention. Reviews and reviews of reviews.

LMICs, low-income and middle-income countries.

### Data extraction from empirical papers

From the included studies, we extracted data (table 2) on: Research design, participant sample, setting, outcome measures; intervention description, components and study duration; Intervention effect; Authors’ programme theory where stated and our interpretation of whether the study worked as intended.

**Table 2:** Data extraction of intervention design and effect, and author’s programme theory from all four included empirical studies on mHealth in Kenya, South Africa, Honduras and Mexico and Pakistan

Author, year and country	Research design, participant sample, setting, main outcome measure and secondary outcome	Intervention description, intervention components and study duration	Intervention effect on primary, secondary and process outcomes and overview	Authors’ programme theory for the intervention (if stated) or probable programme theory, based on description of intervention (including likely mechanism/intervention function)	Did the intervention work as intended or not?
Lester *et al*, 2010^44^: Kenya	Design: RCT Sample: Patients infected with HIV, over 18 years old, initiating ART for the first-time. n=803 total. Setting: One urban university clinic, one urban non-governmental organisation clinic and one rural public health service clinic. Main outcome measure: Self-reported ART adherence (>95% of prescribed doses in the past 30 days); plasma HIV-1 viral RNA load suppression (<400 copies per mL). Secondary outcome: Rate of attrition (not having a final visit at 12 months) and rates of several categories of attrition (mortality, withdrawal from the study, transfer to non-study clinics and loss to follow-up without identifiable cause).	Nurse or clinical officer sends the patients a text message: ‘How are you?’ each Monday morning. Patients asked to respond within 48 hours with: ‘Doing well’ or ‘Have a problem’. Nurse/clinical officer phones the patient who has problems or if there is no response. Patients received intervention training when recruited. Text message sent using multiple recipient bulk messaging. Intervention components: Provision of support communication Observation Reminders Study duration: 12 months (baseline, 6 and 12 months)	Impact outcome: Self-reported adherence: intervention group: 168 (62%); control group: 132 (50%) RR 0.81; 95% CI (0.69 to 0.94); p=0·006 Viral suppression: -intervention group 156 (57%); control group 128 (48%); RR 0.85; 95% CI (0.72 to 0.99); p=0.04. Secondary outcome: No significant associations with the intervention. Intervention group: 19% missing, 9% mortality, 3% withdrawal, 6% loss to follow-up and 1% transferred out. Control group: 23% missing, 11% mortality, 1% withdrawal, 10% loss to follow-up and 0% transfer out (to a different clinic not in the study). Process outcomes: At the end of the study, 191 of 194 patients in the intervention group reported they would like the text message programme to continue, of whom 188 (98%) said they would recommend it to a friend. In the focus group sessions, many patients in the intervention group also reported that they thought the text message support service was valuable. Outcome overview: Significant change in self-reported adherence to ART and suppression of viral loads between groups. Male/urban residence favoured adherence compared with the control group.	The text message acted as an indirect reminder to the patients to take ART and provided support (perceived support was valuable). The patients know they will be followed up if they do not respond, so the design of the intervention acts as an incentive to respond otherwise the nurse or clinical officer will contact them. By receiving a weekly message that asks how they are, the patient is made to feel cared for during the first period they are taking HIV treatment.	**Yes—the feeling of being supported via digital communication was important.** The text message intervention was well received by patients, many of whom reported that they felt ‘like someone cares’. Patients had to reply to the nurse or clinical officer within 48 hours. of receiving the text message, otherwise the nurse will check on them, gave them an incentive to keep on track of replying, which was a quick and straightforward process from their phones. The action of being told they had to respond within a given time initiated the behaviour of the communication between the patient and health worker. **No—there is no actual evidence that the weekly reminder caused the continued or improved adherence in people to take their treatment.** Sharing a phone and being a woman reduced the intervention effect, but these factors are unexplained.
Bobrow *et al*, 2016^45^: South Africa	Design: RCT Sample: Patients with hypertension (SBP<220 mm Hg and a DBP<120 mm Hg) over 21 years. n=1188 total. Setting: One urban large public health service clinic. Main outcome measure: Change in systolic blood pressure at 12 months from baseline measured with a validated oscillometric device. Secondary outcome: Health status measured using EuroQul Group 5-Dimension Self-Report Questionnaire (EQ-5D), proportion of scheduled clinic appointments attended, retention in clinical care, satisfaction with clinic services and care, hospital admissions, self-reported adherence to medication (did they collect their medication) and understanding of basic hypertension knowledge.	*Information-only message intervention:* Automated system sends the patient the text message with predefined messages with health worker’s name at the end of message. Interactivity—weekly, at a time predefined by the participant. Participants received one message per week, either a reminder to attend an upcoming appointment (48 hours prior to scheduled appointment) or a message selected-at-random from the message library. *Interactive message intervention:* Information-only intervention PLUS participants could also select messages allocated to the interactive adherence support and could also respond to selected messages using free-to-user ‘Please-Call-Me’ requests. These requests generated an automated series of responses from the text message delivery system (not a health worker nor a phone call response) offering trial participants options to cancel or change an appointment or change the timing and language of the text messages. Patients also received a text message were sent to either thank participants for attending their appointment or alert participants about a missed appointment 48 hours. postdate. There is no personal contact between nurse and participant. Intervention components: Reminders Targeted actions Study duration: 12 months (baseline, follow-up at 6 months and 12 months).	Impact outcome: The mean (95% CI, p value) adjusted difference in change for the information-only message group compared with usual care was systolic blood pressure −2.2 mm Hg (−4.4 to −0.04, p=0.046) and for the interactive message group compared with usual care −1.6 mm Hg (−3.7 to 0.6, p=0.16). Secondary outcomes: Out of the 86% of patients, the trial had adherence data available, 63% from the information only message intervention group, 60% for the interactive message intervention group and 49% from the usual care group, had 80% of proportion of days covered for blood pressure lowering medication for a 12-month period. EQ-5D scores, attendance at clinic appointments, retention in clinical care, treatment and clinic satisfaction, hypertension knowledge, self-reported adherence, hospital admissions and differences in medication changes did not differ between groups. Subgroup analysis: There was no statistically significant heterogeneity in the treatment effects and there was an indication that active interventions were more effective among older patients (55 years), patients in better control at baseline (<140 mm Hg) and among those with a shorter duration of hypertension (<10 years).	By receiving a behavioural intervention delivered via text message as support, this could improve collection of medicines and may have a small impact on blood pressure as compared with usual care in a general outpatient population of adults with high blood pressure. Getting information to people at a time that is relevant to them and prompts to take medication should help to encourage the patient because they have chosen when to receive the message and this time is most appropriate to their daily routine. Reminder messages with information about forthcoming or missed clinic appointments remind the patient about where they need to be and when. Reminders about who they can contact if they are worried or concerned about any side effects of the medication gives the patient increased knowledge and channels of support. By receiving a ‘Happy Birthday’ message this makes the patient feel cared for by the health service, on their day of birth. Study design issues: The intervention includes behaviour change cluster techniques such as repetition and substitution, goals and planning and social support which are based on Michie’s Behaviour Change Wheel.	**No—the primary outcome did not improve**. Blood pressure levels in both intervention groups did not decrease during the trial period suggesting the text message (information and interactive) did not help to reduce blood pressure control in adults with diagnosed high BP and on treatment (partly because many of them were stable at baseline). **Yes—the secondary outcomes did improve.** Proportion of days of medication covered (adherence to medication) was higher in both intervention groups suggesting that they were remembering to collect their medication because of the reminders: the two intervention groups were collecting the medication more regularly over the control groups.
Piette *et al*, 2012^43^: Honduras and Mexico	Design: RCT Sample: Patients with **hypertension** (‡140 mm Hg if non-diabetic) or hypertension and diabetes* (‡130 mm Hg if diabetic) 18–80 years of age. Intervention group (text message) and preplanned subgroup analysis: patients with low literacy or high BP management information needs (n=89). Control group (standard care) (n=92). *Blood glucose was not being measured in this study. Setting: Four private and two public clinics in Honduras and two clinics in Mexico. Main outcome measure: Change in systolic blood pressure (SBPs ‡160 mm Hg and<180 mm Hg). Secondary outcomes: Patients’ perceived general health status, depressive symptoms (using a validated Spanish 10-item Centre for Epidemiological Studies-Depression Scale), medication-related problems (adherence measured using the Morisky Scale) and satisfaction with care related to hypertension.	Automated telephone monitoring and behaviour-change calls plus home BP monitoring among hypertensive patients. The calls were aimed at gathering information about the patient’s BP, BP self-monitoring, medication adherence and diet and to provide tailored advice based on the patient’s responses. Patients’ with hypertension received a home blood pressure monitor and were given training. The intervention focused mainly on providing information and self-management education to patients via interactive voice calls or automated calls—to check their BP and were asked about recent systolic values above and below the normal range, medication adherence and intake of salty foods. Based on what they said, the patients were then offered additional self-care information during the call and prompts to seek medical attention or medication refills to address unacceptably high or low BP. Structured email alerts for health workers were generated automatically when patients reported that at least half the time in the prior week they had an SBP ‡140 mm Hg (patients with non-diabetes), ‡ 130 mm Hg (diabetic patients) or 100 mm Hg (all patients) or if the patient reported rarely or never taking his or her BP medication or less than a 2 week supply. Patients had the option of enrolling with a family member or friend, who received a brief automated telephone update regarding the patient’s self-reported health status each week, including information about the patient’s hypertension self-care and how that caregiver could help the patient self-manage more effectively. Intervention components: Observation Reminders Motivating education/advice information Study duration: 6 weeks (with option of a 3 month extension).	Impact outcome: Intervention patients’ SBPs decreased 4.2 mm Hg relative to controls (95% CI 9.1 to 0.7; p=0.09). In the subgroup with high information needs, intervention patients’ average SBPs decreased 8.8 mm Hg (–14.2 to –3.4, p=0.002). 57% of intervention patients had controlled BP at follow-up compared with 38% of the comparison group (p=0.006). Process outcome: More than 88% of patients reported that the automated calling system was easy to learn and use, and 93% reported that the automated calls included useful information for managing their hypertension. Overall, 94% of intervention patients reported being very satisfied with the intervention and 76% reported that the programme was excellent. Secondary outcomes: At follow-up, patients had lower depressive scores and fewer medication-related problems for example, worry about the effects of their medication. Overall health was reported and there was greater satisfaction with care-related to hypertension. Subgroup analysis: Patients with low literacy or high BP management information needs: those with problems learning about their health problems because of difficulty understanding written information, had never been told they had hypertension or had not spoken with a clinician about their BP in more than 6 months or were confused about their medication regimen. They had an average of 8.8 mm Hg reduction in SBP relative to controls (95% CI 14.2 to –3.4; p=0.002). Outcome overview: In the overall sample, there was a non-statistically significant (p=0.09) 4.2 mm Hg relative decrease in SBP among intervention patients. In the subgroup of patients with low literacy or high information needs, 8.8 mm Hg reduction in average SBP was observed with a significantly greater proportion of intervention than control patients having BPs in the acceptable range. Intervention patients at follow-up had SBPs that were 4.2 mm Hg lower on average than control patients (95% CI 9.1 to 0.7; p=0.09).	By receiving automated self-management calls, plus home blood pressure monitoring kit, this can improve outcomes for hypertensive patients as reminders to check blood pressure readings several times per week this acts a nudge to action. The mechanisms of action included: (1) During the calls patients were reminded to check their BP regularly and were asked about recent systolic values above and below the normal range, medication adherence, and intake of salty foods. This regular checking-in meant the patient had to keep on top of their management and regularly verbally discuss their chronic disease. (2) The health workers were alerted via email if a patient’s blood pressure changed or if medication was not taken. Therefore, the patients knew that their results were going to be reported if they failed to take their BP medication. This created an incentive to adhere. (3) The support of a family member or friend meant that the patient’s self-reported health status and self-care were given to the treatment supporter. This process meant the patient was accountable to someone else. Another person was part of their chronic disease management. The information-based intervention had greater impact on patients who reported a greater need for hypertension-related knowledge and education because they had low literacy or high information needs and valued additional supportive information.	**Yes—blood pressure decreased**. Calls are effective over a short follow-up period. **Yes—particularly for the subgroups with low literacy and high BP.** The intervention focused on providing information and self-management education to patients.
Kamal *et al*, 2015^42^: Pakistan	Design: RCT Sample: Patients with **stroke** 18 years and over. >1 month since last episode of stroke. Use of at least two drugs such as (but not limited to) antiplatelets, statins and antihypertensives to control risk factors of stroke (n=83). Control group (n=79). Setting: One urban hospital’s neurology and stroke unit. Main outcome measure: Change in self-reported stroke medication adherence after 2 months of receiving the text message (using the Morisky Scale). Secondary outcome: Blood pressure was measured using the Mindray Datascope Equator to detect change in systolic and DBP.	Automated text message reminders customised to each patient’s individual prescription. Patients were required to respond to the text message, stating if they had taken their medicines by replying ‘Yes’ or ‘No’. Also, customised twice-weekly health information text messages were sent according to medical and drug profile of every patient. The timing of the message was decided according to the prescription so that health messages did not collide with reminder messages. Intervention components: Reminders Motivating education/advice information Praise and encouragement Study duration: Baseline, 2 months follow-up.	Impact outcome: Mean difference in adherence score between the intervention group and the control group (usual care) was 0.54 (95 % CI 0.22 to 0.85; p≤0.01). Process outcome: Patient satisfaction and acceptability using text messages to improve clinical outcome, was 96% after the 2-month period. Secondary outcome: Reduction in DBP. No major effect was observed on systolic blood pressure due to reasonably limited exposure time to the intervention. Overview outcome: A significant affect was found in the intervention group’s adherence to stroke medication. It was found that being retired and/or unemployed, being educated and with a higher dosing frequency, were positively related to the level of medication adherence found.	By customising the messages, the patients are likely to be more satisfied to act as they were asked to reply with a very simple response. Text messages were customised for each patient depending on their medication prescription (compared with just receiving simple knowledge transfer messages). The patients were being treated therefore as single cases. Also, the timing of the messages sent out according to the patient’s dosing schedule was important in increasing adherence. This timing targeted an action because they were received at a time appropriate to the person. These messages targeted both intentional non-adherence and non-intentional non-adherence by providing knowledge and belief change messages and other cuing, nudging and reminder behaviours to take medications. Reminders about behaviour were likely to entice the patient to take their medication along with giving praise and encouragement. Study design issues: Content and language of intervention messages are designed on The Health Belief Model and Social Cognitive Theory.	**Yes—in the 2 months studied, adherence improved. There was also a slight reduction in DBP.**

ART, antiretroviral therapy; BP, blood pressure; DBP, diastolic blood pressure; RCT, randomised controlled trial; RR, Relative Risk; SBP, systolic blood pressure.

Where authors did not describe how they thought their intervention would work, we worked this out from careful reading of the description of the intervention. We also referred to related study protocols, if available, or other accompanying papers such as process evaluations of the trial. JAW, FG and KD extracted data independently and then compared data to resolve any inconsistencies.

### Identifying established theory to test the programme theory

We used the CMOc in the diagram in figure 1 and applied this to each empirical study on chronic disease monitoring found in the search.^33^ We developed a summary of how the interventions were intended to work according to the study authors. We then searched for relevant established high-level theory related to study intervention components and through further literature searching. As most of the mechanisms were behaviour-based, we looked mostly to psychology for the theory. We aimed to match established theory to each intervention component type (table 3) to help us reach our final CMOc. The theories helped us to understand how and why each study’s intervention design and possible components of the intervention may have contributed to the study being effective or not. These theories were then used to help us search for higher-level frameworks (table 4) based on similar theories to those described in table 3.

**Table 3:** Intervention components described in relation to established theory in all four included empirical studies on mHealth in Kenya, South Africa, Honduras and Mexico and Pakistan

Components of the intervention	Example to explain the intervention components from study interventions	Established theory that relates to one of more of the intervention components	Types of intervention components used by each study author in the design of their mHealth intervention
Form of communication Two-way communication Whether the communication and response from the health system is automated or not (is there a real person engaging with the patient) Importance of personal contact for motivation and support	This two-way communication which is automated or not may enable the provision of support. Support is the content which flows through the channel of communication and is separate from type of communication. Patient receives tailored digital counselling and feedback after submitting clinical measurements. This enables the establishment of a relationship with health worker and increases the access to the support. The text message or voice call motivates the patient by informing them to eat more vegetables or reduce salt intake. The desire to carry out the behaviour change or adherence to treatment motivated by their beliefs, expectations and feelings.	*Health Communication Theory/Access Framework*^62^: whereby using mHealth enhances access to support from a health worker. *Information-Motivation-Strategy Model*^63^: whereby adherence to treatment requires patients to have (1)Information, (2) Motivation and (3) Strategies to adhere to treatment giving the patient the incentive to behave appropriately and according to their regimen. *Theory of Interpersonal Relationships*^64^: whereby interpersonal interactions between the patient and the health worker can help with task engagement. This theory seeks to understand which characteristics strengthen the bonds between people, encourage empathy and trust and create a sense of well-being after a close interaction to influence human motivation. The stronger the motivation, the stronger the relationship becomes, because empathy and care can derive from the interpersonal processes.	*Two-way communication:* Kamal, Lester, Piette, Bobrow *Automated:* Bobrow, Kamal *Real person:* Lester, Piette
Activities that communication channel is used for: Observation Providing information	*Observation:* Patient is observed indirectly and must regularly send clinical readings. If the patient is being observed this acts as persuasion to maintain monitoring as they are now accountable to someone. *Information:* The text message or voice call contains disease-specific information aimed at increasing knowledge. Increasing patient knowledge through mobile information reminder or learning messages through recommendations.	*Communication for Persuasion*^65^: whereby the patient can be persuaded by a role model whim they feel accountable to. *Health Belief Model*^66^: whereby a patient needs sufficient motivation to make the health issue relevant, the belief it is a serious problem and to follow a recommendation to reduce that threat.	*Observation:* Lester, Piette
Different types of information Reminders Education/advice Targeted actions Praise and encouragement	*Reminders:* Reminder messages to attend clinic appointments to improve retention to care. Reminder messages to take medication to increase adherence. Reminder can act as a cue to action to stimulate the patient into action. Reminders can help to reinforce the patients’ *thoughts and feelings that lead to their decisions (attitude towards behaviour).* *Education/advice:* If a patient listens to educational advice given by a health professional via text or voice, it is possible that if the patient is capable of understanding what they have read or heard, they can then follow the advice to change their behaviour. *Targeted actions:* Digital mobile decision-support is likely to aid the accomplishment of goals. Setting a goal such as taking medication or observing a mood state. *Praise and encouragement:* The text message or voice call is intended to promote good healthily behaviour and making the patient feel optimistic ‘You are doing well’. If a patient receives a call or message about adopting a healthy lifestyle, this promotes a behavioural change.	*Theory of Planned Behaviour*^67^: whereby the patient is made aware of their thoughts and feelings that lead to decisions. *Cognitive Load Theory*^68^: whereby the demands of a certain task on the patient accounts for their own beliefs, expectations and goals on their own load perceptions. *Goal Setting Theory*^69^: whereby the difficulty of the task is mediated because the patient is working towards a goal. Feedback is also useful. *Self-Regulation Theory*^70^: whereby attitude change can result when the patient realises and observes their own behaviours, which can include monitoring their mood.	*Reminders:* Kamal, Lester, Piette, Bobrow *Education/advice:* Piette, Kamal *Provision of support communication:* Lester *Targeted actions:* Bobrow *Praise and encouragement:* Kamal

**Table 4:** Mechanisms of change (RFV and COM-B mechanisms) evident in all four included empirical studies on mHealth in Kenya, South Africa, Honduras and Mexico and Pakistan

RFV and COM-B mechanisms by study author	Intervention components used in each study	Relationships	Fit	Visibility	Capability	Opportunity	Motivation
Lester *et al*^44^ (Kenya)	Provision of support communication, Observation, Reminders	Weekly communication between patient and nurse. Importance of personal contact for motivation.	Text message support service and reminder message as addition to everyday routine with patients who were already phone users.	Receiving the reminder to self-report adherence to HIV treatment increases the awareness of the disease.	Not applicable	If illiterate, the HIV patient must be assisted by literate partner (if willing to disclose their status/and that they are on long term treatment). Respond with that they are doing well or have a problem to the nurses.	Regular communication with health worker motivates patient to remember to take their treatment and keep on top of their disease.
Bobrow *et al*^45^ (South Africa)	Reminders, Targeted actions	Health worker relationship with patient. Just by receiving the message gives the patient feeling someone cares.	Text message as additional reminder system. Function to reschedule appointment automatically via free return text message.	Follow adherence advice from the text message. Text message increased disease awareness, provided tips for health and helped to develop and reinforce more robust reminder systems.	As above—be comfortable with the technology to access and read text- messages.	Opportunity to make changes to clinic times.	Motivation to read the content of the messages and act on it: adhere.
Piette *et al*^43^ (Honduras and Mexico)	Observation, Reminders, Motivating education/advice information	Relationship between patient and the kit sent to the health worker.	Fits into their everyday lives because the kit is at home and is purposely intended for this intervention.	It requires logging blood pressure every day, so it is very regular and this interaction makes the hypertension visible in the person’s life.	The person needs to understand how to use the new technology in their home and the confidence to use the kit and keep using it. They must buy-in to why it is helping them to self-manage.	Allows for the opportunity for social support and a choice to actually receive the support.	Motivates person to reduce salt intake— powerful messages related to behaviours change.
Kamal *et al*^42^ (Pakistan)	Reminders, Motivating education/advice information, Praise and encouragement	Patient must respond to the health worker to inform them they have taken their medication. Patient receives timely, customised messages.	The response is yes or no, so is quick and will not take much of the patient’s time.	By having to take the medication and then action this by sending a message, it creates disease visibility. The information messages ‘take two servings of fruit’ encourage the patient to make healthy choices since they are managing/preventing future strokes.	The patient must have the capability to respond to the cue to action.	The patient must have the opportunity to reply including the comprehension or social support.	The patient must have the motivation to respond to the health worker each day.
Summary of mechanisms that fire to cause/enable change and patient’s engagement in chronic disease monitoring		Intervention must allow for a relationship to form between the patient and the health worker to produce motivation.	Intervention must fit well in the patient’s life and their individual needs.	Intervention makes the disease visible.	Patient need personal capability to respond to the mechanisms of change.	Patient is offered the opportunity for support which may motivate them.	Patient needs motivation to self-monitor.

We found two frameworks to use in our analysis that brought together mechanisms and theories:

- *COM-B framework*,^40^ designed for *Behaviour* change, describes the three domains of: *Capability:* physical skills, knowledge, behavioural regulation and memory, attention and decision process; *Opportunity:* environmental context, resources and social influences; *Motivation:* beliefs about consequences, optimism and beliefs about capabilities and reinforcement and emotion.
- *RFV framework*,^41^ designed for the successful implementation of telecommunications technology health interventions, describes the three domains of: *Relationships:* with health workers and peer as a means of providing support for behavioural change, feedback and reinforcement; *Fit:* integration of mHealth into routine and its ease of use into the existing environment and *Visibility:* to engage in information to mediate and motivate self-management tasks and enable enhanced awareness.

COM-B domains provide a framework for understanding patient behaviours. RFV domains provide a framework to understand how mHealth interventions have been designed to be implemented. The domains of these frameworks include theory relevant to the mechanisms of action of the interventions in our review. We used these theoretical frameworks to understand what mechanisms, in what context will result in a behaviour change.

The Realist and Meta-Narrative Evidence Syntheses (RAMESES I) reporting standards for realist reviews were followed.^33^

## Results

### Search results

**Figure 2:**
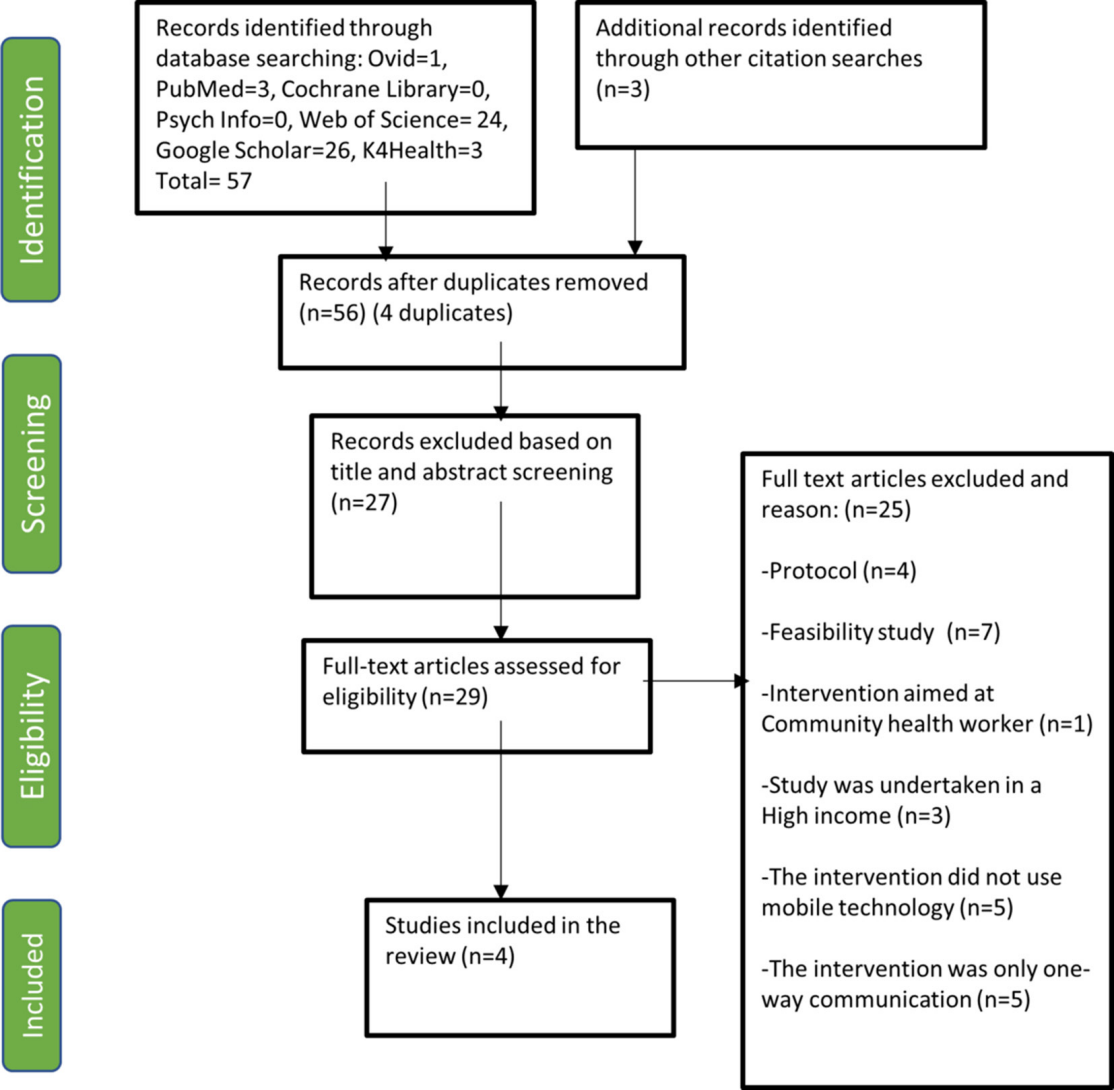
PRISMA.

The process of screening and paper selection resulted in 57 references (PRISMA diagram in figure 2). Of those, 29 papers were assessed for full text eligibility and a total of four studies were eligible for inclusion (table 2).

### The studies and their design

The four papers included studies conducted in Pakistan (Kamal),^42^ Honduras and Mexico (Piette),^43^ Kenya (Lester)^44^ and South Africa (Bobrow).^45^ The studies focused on specific diseases: stroke (Kamal), hypertension (Bobrow and Piette) and HIV (Lester). Patients were all over 18 years of age. All four study designs were randomised controlled trials with control groups receiving usual standard care, all set in urban healthcare settings. Two studies primarily measured clinical outcomes (Bobrow and Piette) and two measured self-reported adherence (Lester and Kamal). The interventions were designed to facilitate communication via mobile phones between the patient and health worker. All interventions involved medication adherence. Two studies had specific subgroup analyses; Piette’s subgroup was low-literate patients with information needs for how to manage high blood pressure; Bobrow’s subgroup was patients over 55 years of age who had good control of blood pressure at the baseline measure and patients on treatment for hypertension for less than 10 years.

The intervention design in three studies was text messages sent weekly to patients (Lester, Bobrow, Kamal). The other study’s intervention was weekly automated phone calls (Piette) (table 2). In two studies, data submitted by patients were interpreted using an automated system (Kamal and Bobrow), whereas in the other two studies, there was engagement with a real health worker (Piette and Lester). In Bobrow’s study, there was an option for the patient to use their phones to initiate a free to user ‘Please Call Me’ request. A health worker did not actually phone the patient back. There was no verbal communication in this instance; instead, this request generated an automated series of responses from the text message delivery system. This design offered trial participants several options including cancelling or moving an appointment, and changing the timing and language of the incoming text messages, whereas in Bobrow’s and Kamal’s studies, the participants predefined when they wanted to receive the message to their phone. This gave them patient choice, as the other studies contacted the patient at a time decided on by the intervention team. In the case of Kamal’s study, when the intervention was described as automated, we had to assume that a health worker was reading the patient’s responses and taking action, especially when the patient had a problem. This was not made explicit in the paper and so it was unclear what the health worker’s role was.

Two studies (Lester and Bobrow), used standardised text messages whereas the other two (Kamal and Piette), used customised messages for each patient (table 2). In Kamal’s study, each patient received tailored text messages with information content about the patients’ individual prescriptions. Likewise, in Piette’s study, the automated calls could be tailored as patients were directly asked to provide blood pressure measurements by using the home monitoring kit, in addition to the patient’s mobile phone. In Lester’s study, they used very indirect ways of assisting patients to monitor as the content of the text messages were aimed at enquiring how they were feeling, without any reference to HIV, whereas in Bobrow’s trial, the information messages did include the words related to the patient’s chronic disease: ‘high blood pills’.

### Intervention components and their intended purpose

Intervention components were similar across the studies, but the combinations of varying components used were different. Intervention components included reminders, patient observation of health state, motivational education/advice, provision of support communication, targeted actions and praise and encouragement. Given the extracted results in table 2, in table 3, we then describe how each intervention’s components relate to theory. We found this midway process useful in helping to define the programme theory.

All except Bobrow’s study required a response from the patient. Instead, the two information groups received information-only messages and the second intervention group also had to the option to interact with the automated message, if desired.

### Programme theory: how the intervention was intended to work

Only Bobrow’s study provided a clear statement of the programme theory and how the authors intended their intervention to work (described in full, in another publication^46^). It was supported by a process evaluation of the study in a paper by Leon.^47^ The other studies mentioned the theories on which they based their intervention design but did not explicitly explain why they felt their intervention worked. For Kamal and Lester’s studies, we used related published study protocols or supplementary material to work out their programme theory. The programme theories are reported in table 2.

Programme theory summary from across the four included studies:

- All four studies used weekly messages/calls. This was intended to make the patient feel cared for. These included non-health related information such as ‘Happy Birthday’ messages (Bobrow).
- In three studies (Lester, Piette and Kamal), a non-response from the patient or the reporting of poor blood pressure readings triggered a health worker to follow-up the patient. This process of follow-up acted as incentive to the patient to always respond in the specified period (Piette).
- In two studies (Bobrow and Kamal), patients were asked, when they wanted to receive the message at a time of day that was relevant to them. This prompted the patient to take action as they had chosen to receive the communication at that specified time.
- In Piette’s study, patients had to take their blood pressure measurements after a cue to action. The other three studies did not require the patient to take clinical measurements as home-monitoring kit was not provided. Also, in Piette’s study, the patient’s report was also sent to a predefined treatment supporter. This was intended to make the patient feel they were accountable to somebody who cares for them. This was the only study to use an external person beyond the patient-health worker.
- Two studies (Piette and Kamal) customised the messages to ensure the patient felt more satisfied and to prompt them into action.
- In two studies, the text messages required simple quick responses such as ‘Yes or No’ (Kamal) or ‘I am fine’ (Lester). These responses were appropriate as the patient did not have to spend much time replying.

### Mechanisms of action

All four studies used the domains of RFV and COM-B as mechanisms of change to some degree (table 4).

### RFV

#### Relationship

There needs to be personal contact within the relationship between the patient and health worker to motivate the patient or the text messages need to be tailored to make the patient feel like someone cares. **Fit:** The level of how well the mHealth intervention fits into the patient’s daily life is crucial to how they will respond. **Visibility:** It appears that visibility acts as a mechanism in the studies because the mHealth component’s purpose is to improve monitoring and to therefore make the disease more visible in the patients’ lives, at least once a week.

### COM-B

#### Capability, Opportunity, Motivation=Behaviour

The interpersonal relationship between the patient and health worker created when the mHealth intervention is not automated improves the patient’s motivation to feel capable of responding or adhering to their treatment regimen. When the patient feels cared for by the real person who is involved, this leads to change in the patients’ behaviour. When the patient is offered the opportunity to be given information that provides education and advice, this may allow them to engage with the information. This in turn may improve the way they manage their disease, as they feel more capable of doing so. Even automated communication can motivate patients to engage.

In all four studies, the patients engage with the intervention components (mHealth resources) by using their own reasoning. Their reasoning is made up of their own motivation and empowerment to respond to the resources. It is this engagement that leads to change and thus improvements in the study’s primary and/or secondary outcomes (table 2, final column). Table 4 summarises the combinations of mechanisms for each intervention.

In Lester’s study, the mHealth components of provision of supportive communication, self-observation and reminders prompted engagement with a health worker (relationship). This personal contact with the nurse resulted in the patient being motivated to collect their medication from the clinic; otherwise a nurse will check-up on them. This personal contact with the nurse and feeling more supported resulted in the patient being motivated to take their treatment (table 4) and keep on top of their disease management. However, this did not lead to improved adherence in treatment.

In Bobrow’s study, the mHealth components were reminders, opportunity to make changes to appointment times so the patient was able to fit the intervention into their daily life. The text messages increase disease awareness and thus its visibility. The mHealth intervention allowed the patient to feel cared for by the nurse and to take more control of their management by having the option to change appointments from their phones. This did not lead to a decrease in blood pressure; however, adherence to medication improved.

In Piette’s study, reminders and motivating education/advice information prompted engagement with a health worker and treatment supporter (relationship). The patient felt they had empathetic support, especially when the patient had problems. Disease management was made more visible as the patient had to log onto the home-monitoring kit every day. Information messages prompted change in lifestyle. This regular interaction resulted in the opportunity to engage with social support when they needed it. By reading powerful practical messages, this motivated them to reduce salt intake for example. This led to a decrease in blood pressure, particularly in the subgroup.

In Kamal’s study the reminders and motivating education/advice information were tailored to the individual patients making the patient feel they had a relationship with their health worker, even though the intervention did not have any direct patient–health worker communication. By having to respond to the health worker, this resulted in the quick ‘Yes’ or ‘No’ response message becoming routinised and did not take much of the patient’s time. This led to improved adherence to medication and a slight reduction in diastolic blood pressure. Table 4 summarises which combinations of mechanisms help to determine what is the outcome of the mHealth intervention.

### Study outcomes

All studies, except Bobrow’s study, achieved their primary outcome. Two studies (Piette and Kamal) had short follow-up periods of 6–8 weeks, respectively. Piette’s intervention was effective in decreasing blood pressure and Kamal’s intervention was effective in increasing adherence to personalised stroke medication. Two studies (Lester and Bobrow) collected follow-up data at 6 months and 12 months. Lester’s study showed effectiveness of self-reported HIV adherence at both 6 and 12 months and improved viral load suppression at 12 months. Bobrow’s study found a non-significant reduction in systolic blood pressure control compared with usual care at both 6 and 12 months. There was significant improvement in their secondary outcome of adherence to collecting medication from the clinic.

### Variation in outcomes by context-related patient factors

In Bobrow’s and Lester’s studies, the interventions were more effective for patients early in their disease trajectory. The interventions are likely to have given the patients opportunity to establish disease management routines. In Piette’s study, the intervention was designed to meet the needs of patients with low literacy and the intervention did have greater effect on this subgroup of patients. The intervention is likely to have increased patient knowledge (from a low level) and so increased opportunity for the patient to engage with managing their disease. The intervention is likely to have challenged beliefs about disease consequences and about their capability to manage their disease leading to increased motivation. Bobrow’s study found that patients who benefited the most from the mHealth intervention were those with high personal stress caused by multiple psychosocial stressors. The intervention is likely to have provided a structure to their disease management that was lacking in other aspects of their life, increasing their motivation to engage with disease management yet without intruding into other aspects of life. We acknowledge there are certain subgroups that respond particularly well to mHealth interventions (and were under subanalyses within the studies) and where their monitoring improves and why this may be so. Patients with low literacy/high information needs who require knowledge and support (any information is better than nothing) and older patients who have good blood pressure control and/or have been diagnosed within the past 10 years.

## Discussion

All the interventions were effective in terms of improving adherence to monitoring of the long-term condition for which they were designed (HIV, hypertension and stroke management). The interventions in the four studies included different combinations of intervention components but each one included a reminder. We were able to map all intervention components to RFV and/or COM-B mechanisms. Some interventions focused more on the relationship aspect of care provision and others on motivating the patient. Subgroup analysis within three studies suggests that contextual factors moderate the impact of the interventions. In the studies that we reviewed, these factors were patient related: low literacy, high personal stress and time since diagnosis. We found no evidence of moderation by non-patient contextual factors.

Our findings are consistent with those of other international reviews (some within high-income settings). Three systematic reviews found reminders for disease monitoring improve healthcare processes but with education through voice call and text messages, both health outcomes and care processes improve.^18 22 23^ A realist review of mobile phone-based health interventions for non-communicable disease management in sub-Saharan Africa^48^ found that patient related contextual factors influenced the impact of interventions. Similarly, text messaging used to improve adherence to antiretroviral therapy had more effect for those with at least primary education.^49^ Taking account of sociocultural factors has been shown to influence scale-up and sustainability of mHealth interventions.^50^ Reviews have found variation in effect depending on how mechanisms were operationalised. For example, weekly reminders worked better than daily reminders, and messages had more impact when they were linked with patient–health worker interaction.^17 27 49 51–53^ In our review, we were unable to identify variation in impact related to how the mechanisms were operationalised. Our review found no studies where patients received test results or instructions to adjust medication. In high-income countries, interventions have included these aspects of care.^18 22 23^ Most reviews of use of digital communication with patients in sub-Saharan Africa, comment on the problems of implementation of the communication system, particularly technical and maintenance issues.^26–28 54–58^ In our included studies, implementation issues had been resolved.

### Strengths and limitations

A realist approach was methodologically appropriate for our research question. However, we were limited by the design and reporting of the included studies. We do not know whether all of the study authors were aware if their intervention designs included domains of RFV and COM-B as mechanisms of change. Among our included studies, there were no interventions of sufficient similarity to allow comparison of outcomes when used in different contexts. Within each study, the authors did not describe variation in the context in which the intervention was delivered. This limits our ability to critically examine the programme theory and could weaken the explanatory power of our conclusions.

The lack of detail in the included study interventions may mean we incorrectly interpreted how and why the intervention worked. Only one of the three studies included a process evaluation. Interviews with the study authors would have clarified their programme theory for their intervention. This would possibly deepen our analysis by accounting for the socioecological dimensions of behaviour rather than being individually focused. The studies had limited follow-up over time, so sustainability of intervention use and sustainability of effect is unclear. The lack of evidence on sustainability is recognised as a serious problem in mHealth pilots in low-resource settings.^59^ The only contextual factors we identified were those measured as patient characteristics and used in subgroup analyses. Also, we may have missed papers for inclusion if we had included non-English papers and extended our search to nursing databases.

### Policy and practice implications and future research

Our review suggests there is potential for improving the monitoring of chronic disease in LMICs using two-way digital communication, although close connection with local care provision is important. Behavioural theory can guide the design of mHealth interventions aimed at changing health behaviours.^60^ Wong *et al*^33^ states that realist reviews reveal ‘what policy makers or practitioners might put in place to change the context or provide resources in such a way as to most likely trigger the right mechanism(s) to produce the desired outcome’. It is important to target interventions where impact is most likely, for example, when a patient has more recently been diagnosed. Other contexts which information based mHealth may contribute are when patients have low literacy and low knowledge about their disease. Patients who are under stressful life situations such as poverty, family bereavement or managing multiple long-term conditions may also be amenable and respond well to mHealth support.

Further research is needed on the tailoring of mHealth interventions to the needs and sociocultural context of the patient. For example, the *STAR2D study^61^ underway has found that tailoring dietary advice to patient context is appropriate.^46^

## Conclusion

Although chronic disease management is a major burden on healthcare providers in low-resource contexts, there is very little evidence for the use of mHealth for improving chronic disease monitoring in these settings and what exists is mainly on medication adherence. The interventions that have been evaluated mapped to established behaviour theory though this was not always explicit in their description of their mHealth design. There was little evidence of how context moderated the effect of the intervention, except for contextual factors reflected in patient characteristics such as low literacy, under stress or being early in the disease trajectory.
